# Investigation of the binding properties of a multi-modular GH45 cellulase using bioinspired model assemblies

**DOI:** 10.1186/s13068-016-0428-y

**Published:** 2016-01-19

**Authors:** Monica Fong, Jean-Guy Berrin, Gabriel Paës

**Affiliations:** UMR0614, Fractionnement des AgroRessources et Environnement, INRA, 2 esplanade Roland-Garros, 51100 Reims, France; UMR0614, Fractionnement des AgroRessources et Environnement, University of Reims Champagne Ardenne, 2 esplanade Roland-Garros, 51100 Reims, France; UMR1163, Biodiversité et Biotechnologie Fongiques, INRA, 13288 Marseille, France; UMR1163, Biodiversité et Biotechnologie Fongiques, Aix Marseille Université, 13288 Marseille, France; UMR1163, Biodiversité et Biotechnologie Fongiques, Polytech’Marseille, 13288 Marseille, France

**Keywords:** Lignocellulose, Model assemblies, Affinity, Interaction, Cellulase, Carbohydrate-binding module, Modularity

## Abstract

**Background:**

Enzymes degrading plant biomass polymers are widely used in biotechnological applications. Their efficiency can be limited by non-specific interactions occurring with some chemical motifs. In particular, the lignin component is known to bind enzymes irreversibly. In order to determine interactions of enzymes with their substrates, experiments are usually performed on isolated simple polymers which are not representative of plant cell wall complexity. But when using natural plant substrates, the role of individual chemical and structural features affecting enzyme-binding properties is also difficult to decipher.

**Results:**

We have designed and used lignified model assemblies of plant cell walls as templates to characterize binding properties of multi-modular cellulases. These three-dimensional assemblies are modulated in their composition using the three principal polymers found in secondary plant cell walls (cellulose, hemicellulose, and lignin). Binding properties of enzymes are obtained from the measurement of their mobility that depends on their interactions with the polymers and chemical motifs of the assemblies. The affinity of the multi-modular GH45 cellulase was characterized using a statistical analysis to determine the role played by each assembly polymer. Presence of hemicellulose had much less impact on affinity than cellulose and model lignin. Depending on the number of CBMs appended to the cellulase catalytic core, binding properties toward cellulose and lignin were highly contrasted.

**Conclusions:**

Model assemblies bring new insights into the molecular determinants that are responsible for interactions between enzymes and substrate without the need of complex analysis. Consequently, we believe that model bioinspired assemblies will provide relevant information for the design and optimization of enzyme cocktails in the context of biorefineries.

**Electronic supplementary material:**

The online version of this article (doi:10.1186/s13068-016-0428-y) contains supplementary material, which is available to authorized users.

## Background

Plant biomass is considered as a renewable feedstock that could provide fuels, chemicals, and materials, thus decreasing our dependency on fossil resources and limiting climate change. The key challenge today is to deconstruct plant biomass efficiently. Indeed, plant biomass is a highly complex matrix made of polysaccharides (cellulose and hemicellulose) and phenol-based polymers (lignin). They are connected to each other by numerous covalent and non-covalent interactions, to make the so-called lignocellulose. In nature, filamentous fungi are able to deconstruct efficiently these polymers using an arsenal of hydrolytic and oxidative enzymes [1, 2]. In particular, carbohydrate active enzymes (CAZymes) can have various modular organizations with catalytic domains and one or more non-enzymatic modules such as carbohydrate-binding modules (CBMs), which help targeting enzyme substrates [3, 4].

Fungal enzyme cocktails are used industrially to convert plant biomass into sugar monomers subsequently fermented into bioethanol as fuel. Even if some industrial plants have been recently set up [5], the process is still not optimized, in part because enzyme activity is impeded by several features, resulting in high operating costs. These features are generally categorized as those limiting enzyme accessibility to substrate by steric hindrances (physical constraints) and those inactivating enzymes through non-specific interactions, keeping them far from their substrate, or inhibiting enzymes (biochemical constraints).

In order to facilitate the enzymatic hydrolysis step, industrial processes preferentially use pre-treatments to “soften” plant materials. Depending on the type and severity of pre-treatments, plant polymers are more or less chemically altered. For example, some lignin-carbohydrate complex (LCC) droplets appear after dilute acid pre-treatment and migrate through plant cell walls [6]. Cellulose then becomes more exposed but delocalized lignin is able to bind to enzymes and the inhibitory effect of lignin can counterbalance the opening of the plant cell wall [7]. This effect seems increased when pre-treatment severity is high [8]. Since mechanisms by which lignin limits enzyme hydrolysis are important to unravel, many studies have been performed. Analysis of industrially pretreated substrates has shown that cellulases are irreversibly lost when they become adsorbed onto lignin-derived materials [9, 10]. This is confirmed by the measurement of interaction forces by atomic force microscopy: interactions are stronger between cellulases and lignin than between cellulase and cellulose [11]. Microscopic observations using PEG have demonstrated that solvent-exposed lignin is likely to be responsible for non-productive binding of enzymes [12] so that addition of PEG during saccharification increases glucose yields [13] by keeping cellulases free. Different spectroscopic analysis, surface accessibility, and adsorption measurements have been carried out to correlate the presence of chemical motifs belonging to different biomass samples with the binding of enzymes [14, 15], but data are difficult to interpret. Indeed, mechanisms occurring during pre-treatments are not well understood at the molecular level. Molecular modeling approaches have mimicked the composition and organization of the secondary plant cell walls [16]. Proposed models can help to appreciate the relative distribution and association patterns between polymers depending on pre-treatments [17], showing for example that lignin has a tendency to associate more easily with crystalline cellulose than amorphous cellulose. Overall, lignin is well known to limit enzyme activity but the exact mechanism by which it operates is still a matter of debate.

In order to select the most appropriate enzymes to be used to hydrolyze plant materials, not only their activity but also their affinity must be evaluated. Recently, a versatile high-throughput analysis using chromogenic substrates has been set up to measure activity of lignocellulose-active enzymes [18], which is a substantial step forward. Determination of binding properties is usually performed with chemically simple polymers or oligomers not relevant because these ligands are not representative of the plant cell wall architecture. To address this question, we have devised bioinspired assemblies containing different polymers isolated from plant cell walls [19]. These assemblies are based on feruloylated arabinoxylans (FAXs), which are the most abundant hemicellulose of grass secondary cell walls [20] available in large quantities in agricultural residues (e.g., straws and leaves). Ferulic acids (FAs) on FAXs are of primary chemical importance in the architecture of unlignified plant cell walls by creating di- and multi-ferulic linkages [21]. In the bioinspired assemblies, the role of FAs consists in the creation of intra- and inter-FAX cross-linkages leading to the formation of gels [22]. Cellulose nanocrystals (CNCs) can also be incorporated before triggering gelation.

In the lignocellulose plant cell wall, lignin makes connections with hemicellulose through non-covalent and covalent bonds, called LCCs. Lignin is a complex polymer made of three different monolignols: *p*-coumaryl (S-unit), coniferyl (G-unit), and sinapyl alcohols (H-unit) [23] and it generally accounts for 25–35 % in weight of dry plant lignocellulose [24]. Also, hemicellulose and lignin create a dense matrix surrounding cellulose microfibers. Lignin is thus considered as the key component blocking enzymes by preventing enzyme accessibility and by binding enzymes due to its hydrophobic properties [25, 26]. In order to complete these model assemblies, model lignin (dehydrogenation polymer, DHP) was added into the system. As a result, these assemblies can be considered as plant cell wall bioinspired systems with relevant properties as compared to isolated polymers or complex biomass. They are easy to design (polymer type and concentration), to prepare and to characterize; they display a three-dimensional architecture and contain some chemical features identical to those found in plant cell walls.

Model assemblies have been previously used to model the diffusion of fluorescent probes [27] and to measure the affinity properties of some CBMs [28]. In this study, we have investigated the interactions of a set of modulated endoglucanases from glycoside hydrolase family 45 (GH45) appended or not to one CBM or five CBMs from family 1 [29]. Endoglucanases are of primary importance enzymes involved in the degradation of cellulose. These enzymes have been labeled with a fluorophore to obtain fluorescent probes and further embedded into the model assemblies. Mobility of the probes was assessed using the fluorescence recovery after bleaching (FRAP) technique [30, 31] and turned into binding information. Several features related to the enzymes (number of CBMs) and to the assemblies (polymer type and concentration) were analyzed using a full statistical analysis of the dataset to assess the impact of each component on the enzyme-binding properties.

## Results and discussion

### Characterization of the bioinspired assemblies

**Fig. 1:**
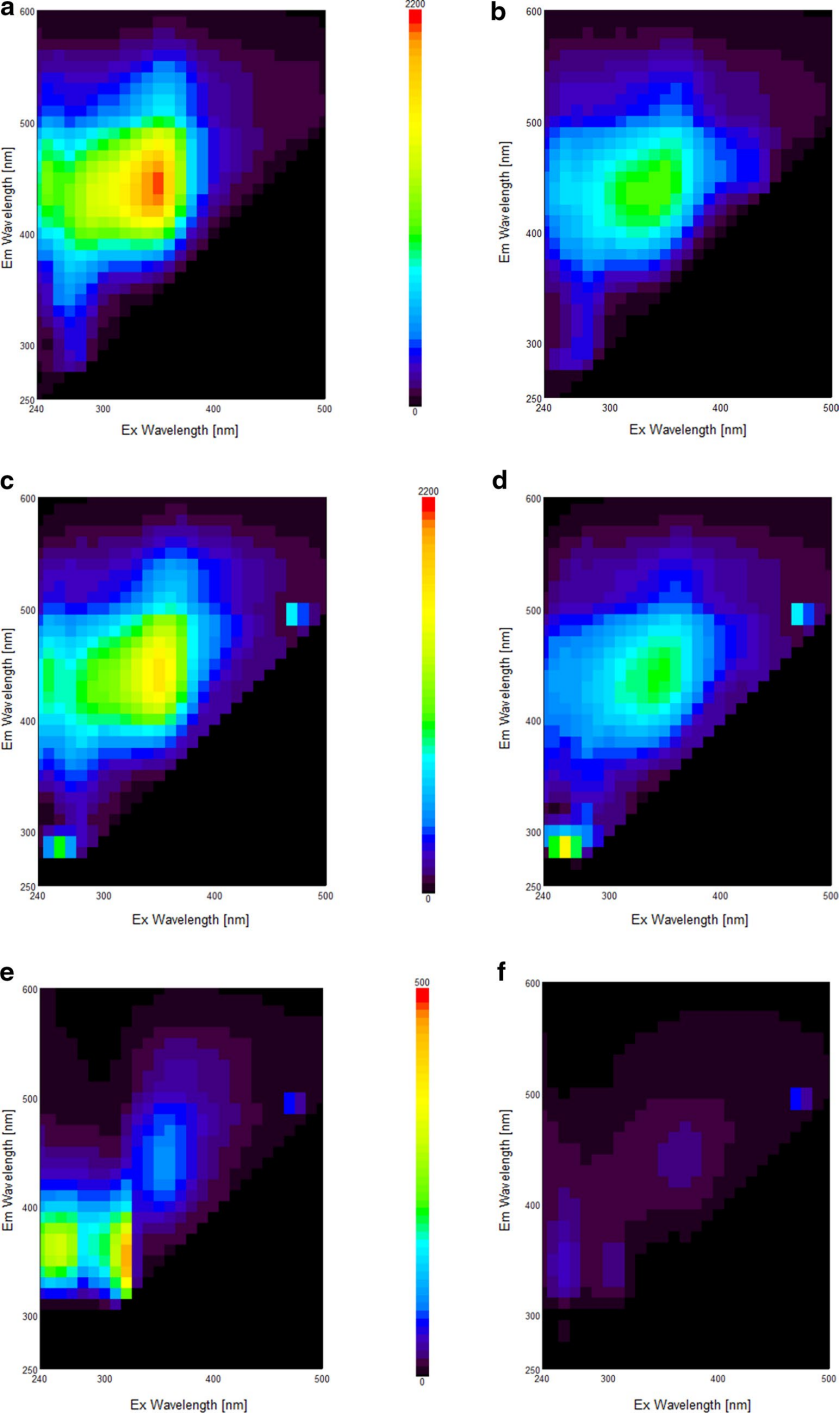
Fluorescence spectra of the assemblies before (*left*) and after (*right*) gelation. **a**, **b** 0.5 % FAX assemblies; **c**, **d** 0.5 % FAX + 1.0 % CNC assemblies; **e**, **f** 0.5 % FAX + 1.0 % CNC + 0.2 % CA assemblies. Fluorescence intensity is an arbitrary unit

Gelation of different assemblies varying in their polymer composition was first followed by spectrofluorescence. 3D spectra were measured at two different time points for three representative assemblies containing 0.5 % FAX/0.5 % FAX + 1.0 % CNC/0.5 % FAX + 1.0 % CNC + 0.2 % CA: before triggering gelation and 2 h after gelation had started (Fig. 1). In the 0.5 % FAX assembly (Fig. 1a), maximum fluorescence appeared at *λ*_ex_ = 350 nm and *λ*_em_ = 450 nm, which corresponds mainly to the FA fluorescence from the FAX [32]. After gelation (Fig. 1b), a strong bathochromic effect was observed since maximum fluorescence intensity was decreased by more than 50 %, while excitation and emission maxima were not altered. In the 0.5 % FAX + 1.0 % CNC assembly (Fig. 1c), the maximum fluorescence intensity had the same *λ*_ex_ and *λ*_em_, but intensity was decreased by 30 % in comparison to the assembly without CNC. After gelation (Fig. 1d), maximum intensity was also decreased by CA 50 %. The large fluorescence reduction demonstrates that the FA fluorescence is masked when FAX chains are cross-linked. This probably results from π–π stacking interactions between FA and from their chemical modifications when they are connected to each other as di-FA [32].

Contrary to the two prior assemblies, the ones containing 0.2 % CA had a very different profile (Fig. 1e). First, several fluorescence maxima were present: one at *λ*_ex_ = 350 nm/*λ*_em_ = 450 nm seen previously, and two other ones at *λ*_ex_ = 260 nm/*λ*_em_ = 360 nm and *λ*_ex_ = 320 nm/*λ*_em_ = 360 nm. Moreover, intensity of the first maximum was drastically reduced by more than 90 % compared to that of the assembly containing only 0.5 % FAX. After gelation (Fig. 1f), fluorescence intensity of the three maxima was also decreased by more than 95 %. This means that FA and CA correspond to distinct fluorescence spots, which were drastically reduced after gelation. Not only the cross-linking reaction of FAX but also the polymerization of CA into DHP are responsible for this observation. Both compounds probably also react to create pseudo LCCs [33]. Similar results were obtained with assemblies displaying higher concentrations of FAX and CNC.

**Fig. 2:**
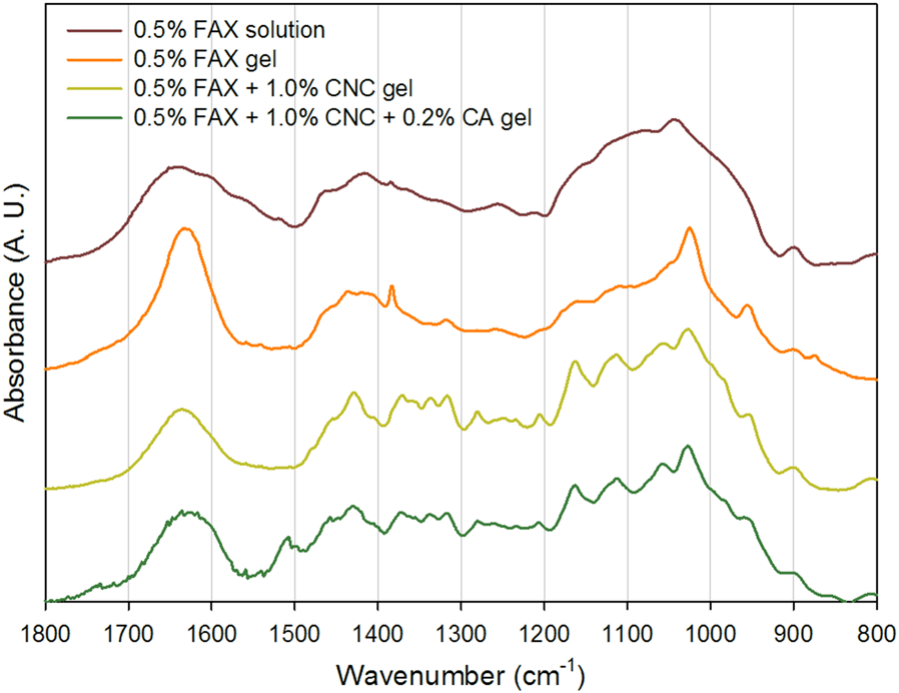
FT-IR of representative assemblies of increasing complexity

In order to complete spectrofluorescence characterization, FT-IR analyses were performed on the same assemblies (Fig. 2). Comparison of FAX and cross-linked FAX showed that bands typical of glycosidic linkages, found in arabinoxylan between 1100 and 1000 cm^−1^, were very narrow in gel assemblies, indicating that the polymer chains were much more constrained in gels. The band at 1630 cm^−1^ was also narrower in FAX gels than in non-cross-linked FAX. This originates from the elongation vibration between aromatic molecules, such as covalently connected FAs. When CNC was added to FAX in gels, a few bands typically corresponding to cellulose appeared: 1160 cm^−1^ for the C–O–C asymmetrical stretching and 1280 cm^−1^ for the C–H bending in crystalline cellulose like CNC [34]. More interestingly, the addition of CA led to the apparition of two new bands: 1508 cm^−1^ typical of aromatic skeletal vibration of lignin and of guaiacyl units in particular [35] and 1460 cm^−1^ corresponding to the asymmetric C–H bending from the methoxy group such as CA forming DHP [36].

Overall, characterization of the assemblies revealed that the FAX polymers were cross-linked and that CA polymerized into DHP. Moreover, covalent interactions between FA and DHP may have been created suggesting that CNCs were trapped into a hemicellulose-lignin matrix.

### Mobility of probes into FAX/CNC assemblies

**Fig. 3:**
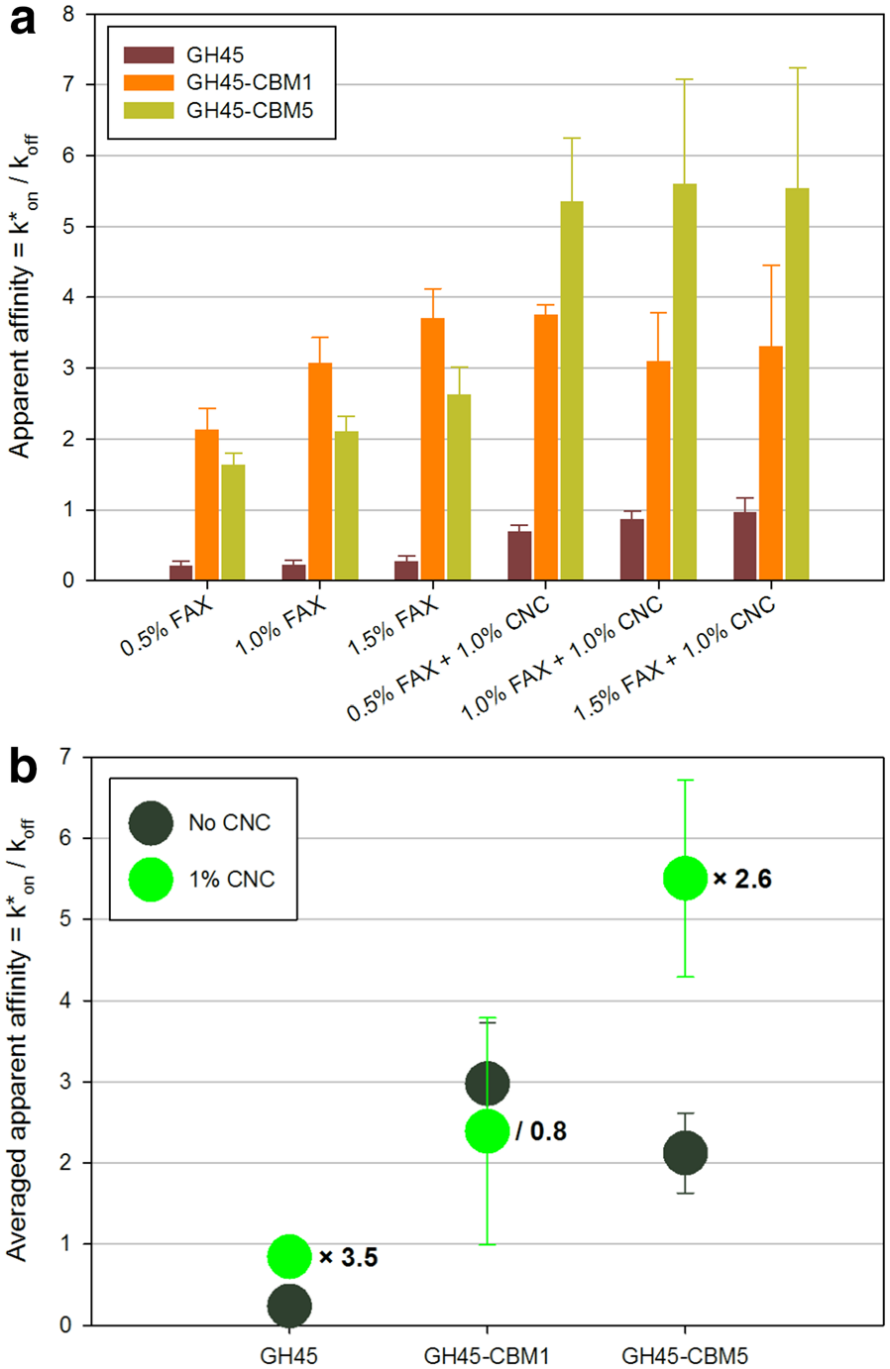
**a** Apparent affinity constants of GH45 probes in FAX/CNC assemblies. *Error bars* are the standard deviations (*n* = 5). **b** Influence of the presence of CNC on apparent affinity of GH45 probes in FAX + CNC assemblies. The number next to the symbols indicates the ratio of averaged apparent affinity between 1 % CNC and no CNC. *Error bars* are the standard deviations (*n* = 5)

The three different GH45-derived probes were first embedded into FAX/CNC assemblies in which FAX concentration was 0.5–1.0–1.5 % and CNC concentration was 0–1.0 %. Diffusion of the probes was assessed in the six different assemblies (data not shown) and the apparent affinity was derived from these measurements (Fig. 3a). For the GH45 probe, affinity was low in FAX-only assemblies and the FAX concentration had no influence. When CNC was added, the affinity was increased by three–fourfold. The two other probes GH45-CBM1 and GH45-CBM5 had a very different behavior. Indeed, their affinity values were much higher than that of GH45 in FAX-only assemblies, and they increased with FAX concentration, GH45-CBM1 having an unchanged affinity. The presence of CNC had a drastic impact on GH45-CBM5 whose affinity was more than doubled, whereas that of GH45-CBM was unchanged. To get a better view of the influence of the CNC concentration, Fig. 3b shows the averaged affinity values for each probe. This clearly confirms that GH45 is largely influenced by the presence of CNC, like GH45-CBM5. Inversely, the probe displaying only one CBM1 module (GH45-CBM1) was very modestly influenced by the presence of CNC.

To quantify the influence of each modulated parameter, the affinity data were exploited to design a full factorial experiment used to model mobility of fluorescent dextrans and CBMs in bioinspired assemblies [27, 28]. In this framework, pseudo-affinity constants of each probe are called responses, influenced by the three modulated parameters that are named principal factors *X*_*n*_ (*n* = 1–3) and defined at two or three levels based on their modulation. Each principal factor was associated with a principal coefficient, A (probe type), B (FAX concentration), and C (CNC concentration), and interaction coefficients AB, AC, BC, and ABC. F-values of each of these coefficients were calculated, and only significant coefficients have been displayed (Additional file 1: Fig. S1). Coefficient A has the highest impact, more than twice that of coefficient C, while coefficient B is the lowest, resulting in the order A > C > B with the ratio of 11:4:1. Interestingly, the only significant interaction coefficient is AC, corresponding to the interaction between the coefficients presenting the highest impacts (probe type and CNC concentration).

### Mobility of probes in FAX/DHP assemblies

**Fig. 4:**
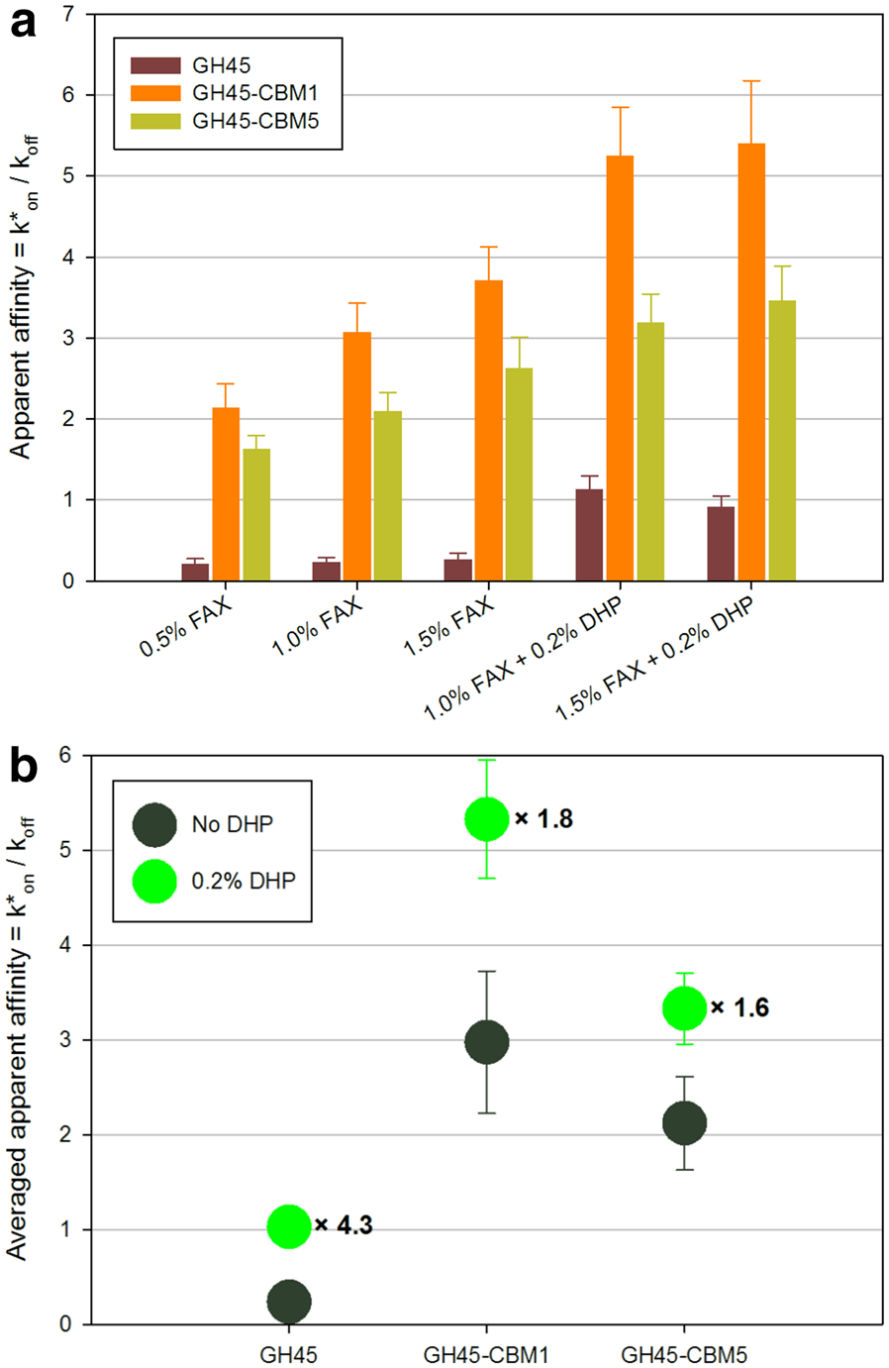
**a** Apparent affinity constants of GH45 probes in FAX/DHP assemblies. *Error bars* are the standard deviations (*n* = 5). **b** Influence of the presence of CNC on apparent affinity of GH45 probes in FAX/DHP assemblies. The number next to the symbols indicates the ratio of averaged apparent affinity between 0.2 % DHP and no DHP. *Error bars* are the standard deviations (*n* = 5)

The same strategy was applied to assemblies containing FAX and DHP. FAX concentration was 0.5–1.0–1.5 % as previously, while DHP concentration was 0–0.2 %. Even with such a low DHP concentration (fivefold less than CNC concentration in the previous assemblies), some significant effects could be observed (Fig. 4a). The maximum increase was observed for the GH45 probe, while GH45-CBM1 and GH45-CBM5 were notably influenced but less than GH45. The changes in FAX concentration when DHP was added did not make any difference in probe affinity. Figure 4b shows that, on average, GH45 is the most influenced probe, even if its affinity remains the lowest. Importantly, all the probes were influenced by the presence of DHP. The full factorial experiment here was performed with 3 parameters whose principal coefficients were A (probe type), B (FAX concentration), and C (DHP concentration). F-values indicate that the coefficient influences are in the order A > C > B with the ratio 28:8:1 with no significant interaction coefficients (Additional file 2: Fig. S2). The presence of DHP is critical regarding affinity, but DHP influence depends on the type of probe, in a larger extent than the influence of CNC.

### Mobility of probes in FAX/CNC/DHP assemblies

**Fig. 5:**
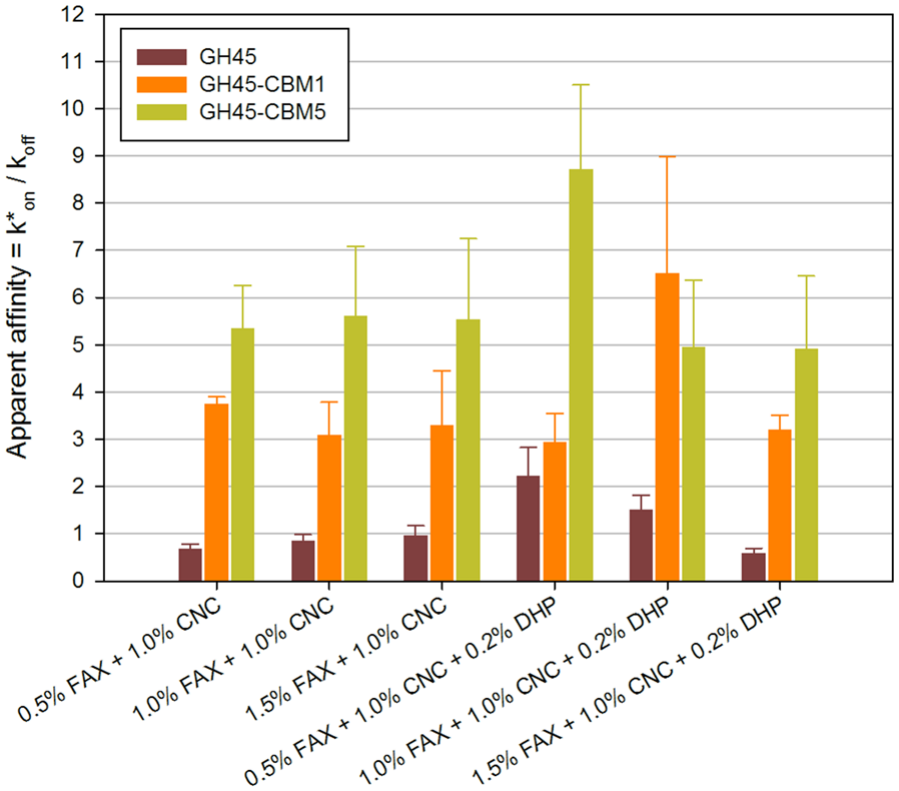
Apparent affinity constants of GH45 probes in FAX/CNC/DHP assemblies. *Error bars* are the standard deviations (*n* = 5)

In the last series of measurements, all the polymers were mixed, by maintaining CNC concentration at 1.0 % and varying FAX concentration at 0.5–1.0–1.5 % and DHP concentration at 0–0.2 %. For GH45 probe, the affinity was mostly increased when DHP was present and FAX concentration was 0.5 %, then decreased with higher FAX concentration (Fig. 5). For GH45-CBM5, the profile was roughly the same: the highest affinity with DHP and low FAX concentration. But for GH45-CBM1, maximum affinity was measured in assemblies with DHP and FAX at 1.0 %. Statistical analysis again involved three parameters whose principal coefficients were A (probe type), B (FAX concentration), and C (DHP concentration). F-values indicate that they are in the order A > C > B with the ratio 40:5:1 (Additional file 3: Fig. S3). This means that the type of probe is by far the most critical parameter (eightfold more compared to the DHP concentration), as seen previously.

**Fig. 6:**
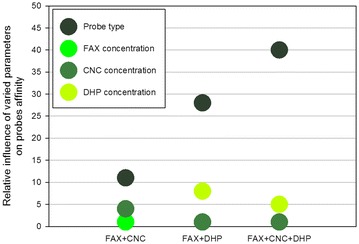
Comparison of the relative influence of the four parameters modulated (probe type and FAX, CNC and DHP concentrations) in the three assemblies

Overall, the relative influence of the parameters modulated on probes affinity can be summarized for each kind of assembly tested (Fig. 6). FAX concentration is the parameter with the least impact on affinity. Indeed, even if FAX is responsible for creating the scaffold of the assemblies and connecting the other polymers, this is not a ligand or substrate for GH45 enzymes which preferentially interact with cellulose [29]. CNC concentration has also a low relative impact on affinity, whereas DHP concentration has a higher impact than FAX and CNC concentrations. But the influence of these three parameters is minor compared to the influence of the probe type, in particular when DHP is present. A deeper analysis of probe modularity is necessary to fully understand their affinity behavior.

### Significance of GH45 modularity on affinity

**Fig. 7:**
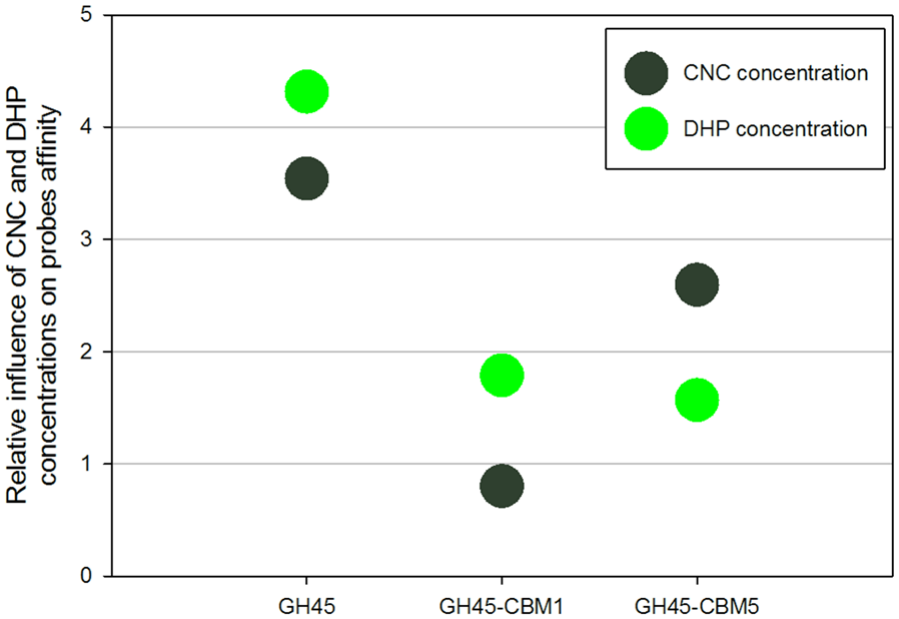
Comparison of the relative influence of CNC and DHP concentrations on each probe affinity

Gathering data from Figs. 3b and 4b, the influence of the two most important parameters, CNC and DHP concentrations was drawn for each probe (Fig. 7). It is important here to make the difference between the influence of one parameter on affinity and the measurement of the affinity by itself. For instance, GH45 has an overall lower affinity than other probes, whatever the type and concentration of polymers are. But GH45 is the probe whose affinity underwent the most severe changes due to the presence of DHP and CNC. GH45-CBM1 follows the same trend as GH45, but CNC and DHP concentrations have a lower influence. The influence of DHP on GH45-CBM5 and GH45-CBM1 is comparable. The striking difference concerns CNC whose influence is much higher for GH45-CBM5 than for GH45-CBM1. So interestingly, even if they all have in common the same catalytic module, the affinity of the three probes was variously influenced by the presence of CNC and DHP, and this clearly depends on their modularity, i.e., the presence or not of one or several CBMs.

From the literature, CBMs are known (1) to increase the effective enzyme concentration on the substrate surface by targeting some structural motifs, resulting in an enhancement of hydrolysis of insoluble substrates [4] and (2) to disrupt the crystalline substrate by weakening and splitting hydrogen bonds, which is part of the more global substrate amorphogenesis [37]. Different studies have previously shown the importance of the number of CBMs for enzyme activity on crystalline cellulose [38] and for enzyme affinity [39]. Nature and length of the linkers connecting the catalytic domain to the CBM or connecting the CBMs between each other can also be important. Linker glycosylation can be critical [40], while flexibility can affect a GH45 cellulase catalytic efficiency [41]. CBMs appended to cellulases and in particular CBMs from family 1 are known to target crystalline cellulose thus facilitating enzymes/substrate interaction [3] and enhancing hydrolysis of crystalline cellulose [42]. Consequently, removal of CBMs from cellulases reduces their hydrolytic activity on insoluble crystalline substrates, whereas their activity remains unchanged on soluble substrates.

Regarding the interactions of GH45 probes with CNC, they can occur with the catalytic module and the family 1 CBMs. The presence of CNC in the assemblies mainly increased interactions of GH45 (by fourfold) and GH45-CBM5 (by nearly threefold), while that of GH45-CBM1 was not altered (Fig. 7). The single GH45 catalytic module is rather small (*R*_H_ = 2.5 nm, Table 1) and can diffuse easily in the assemblies. Its large solvent-exposed surface also facilitates interactions with chemical features of the assemblies, like CNCs. Comparatively, GH45-CBM5 is much larger (*R*_H_ = 7.4 nm, Table 1), which limits its diffusion. Indeed, we have previously shown that even small differences in *R*_H_ between molecular probes lead to important changes in diffusion in FAX + CNC assemblies [19, 27]. But the large size of GH45-CBM5 may be counterweighted by the presence of 5 CBMs having a natural affinity for cellulose. GH45-CBM1, even if bearing only one CBM, is twice as large as GH45 (*R*_H_ = 4.6 nm, Table 1): it does not benefit from the large array of CBMs of GH45-CBM5, and it is not small enough to diffuse easily like GH45.

**Table 1:** Biochemical properties of the probes

	Theoretical molecular weight (kDa)	Experimental molecular weight (kDa)	Experimental hydrodynamic radius *R*_H_ (nm)	Degree of FITC labeling
GH45	23	25.0	2.5	1.0
GH45-CBM1	31	47.0	4.6	1.0
GH45-CBM5	62	79.4	7.4	1.0

Theoretical values are based on protein sequences (see Additional files 1, 2, 3, 4), experimental values are based on light scattering data

These conclusions can seem contradictory with the results from our previous study [29]. Binding to crystalline cellulose (Avicel PH101) was substantially boosted by the presence of one CBM rather than five, so the presence of five CBMs in GH45-CBM5 was not advantageous. To explain this observation, our hypothesis was that the steric hindrance of five CBMs could prevent the exhibition of the flat surface required for interaction with cellulose chains. Upon deletion of four CBMs, the enzyme probably recovered an extended conformation more adapted to efficient cellulose binding. Actually, two different types of crystalline cellulose have been used. CNC (this study) and Avicel PH101 (previous study) are both crystalline cellulose products obtained from mineral acidic hydrolysis of cellulose, but the process conditions used give them different properties. CNCs are nanoparticles of pure crystalline cellulose consisting of 100-nm-long rods [43]. Avicel PH101 crystalline cellulose is, in comparison to CNC, made of agglomerated microcrystals shaping large particles (50 µm) (provider data). So, while CNCs expose only flat and homogeneous surfaces, Avicel PH101 is made of porous homogeneous particles. Recognition mechanisms of such different substrates must of course be different. Comparison of binding properties of GH45 probes on these substrates demonstrates that they do not behave the same way.

Regarding the interactions with DHP, it is important to stress that even at a final concentration as low as 0.2 %, which is fivefold lower than that of CNC, DHP demonstrates an important sticking effect by limiting diffusion of the probes and increasing their interactions. This result is in agreement with the role played by lignin regarding its strong affinity for enzymes leading to an irreversible protein adsorption [14, 15] and enzyme inactivation [9, 44]. In our model assemblies, GH45-CBM1 is the most affected probe, surprisingly more than GH45-CBM5 and GH45 (Fig. 7). This means that the chemical protein features which bind to DHP are more accessible in GH45-CBM1, so probably localized in its CBM. Different studies have suggested that non-productive enzyme binding on lignin might be due to hydrophobic interactions mediated by CBMs, thus decreasing cellulose hydrolysis [44]. Other studies, though, have demonstrated that CBMs did not contribute to non-productive adsorption [45]. Usually, these interactions are driven by hydrophobic interactions and occur between tryptophan residues and polymer sugars [46]. Based on the sequence analysis of the three GH45 probes (Additional file 4: Fig. S4), the catalytic module possesses 5 Trp residues, while GH45-CBM1 and GH45-CBM5 have 2 and 9 additional ones, respectively. But in order to bind to DHP, Trp residues also must be solvent accessible. Usually, such aromatic residues, in particular in CBMs, are necessarily close to the surface. However, they can become masked by other elements of the proteins such as the linker or glycosylation motifs mainly located in the linker regions. Difference between theoretical and experimental MWs (Table 1) indicates that glycosylation increases MW by 50 % and 30 % for GH45-CBM1 and GH45-CBM5, respectively. From our data and known binding behavior of CBMs, we can postulate that the aromatic residues binding to DHP are located close to the *N*-terminal end of the first CBM and become masked when other CBMs are attached like in GH45-CBM5. So, in the case of GH45 cellulase, the presence of 5 CBMs instead of only one is advantageous, since it limits non-specific interactions with lignin, which was not predictable at first glance. This hypothesis could be checked by performing specific mutations of Trp residues close to the *N*-terminal end which seems relevant mutagenesis targets to reduce interaction with DHP. Swapping CBMs to the *C*-terminal region instead of the *N*-terminal could also be an interesting approach.

## Conclusions

In the biorefinery processes, the use of enzymes as catalysts provides many advantages such as substrate selectivity and less energy consumption. But inactivation and inhibition of enzymes are still problematic. It is, therefore, critical to better understand their behavior when interacting with their substrate. The design of lignified assemblies to assess the binding properties of modular cellulases has revealed that the role of CBMs varies depending on the type of substrate considered. The presence of 1 or 5 CBMs could lead to opposite binding effects, which can be advantageous or detrimental, indicating that binding is not necessarily dominated by CBM. Binding interactions at the molecular level should be further investigated to pinpoint the molecular determinants and polymer motifs that interact between each other. Overall, we believe that model bioinspired assemblies provide relevant information for the design and optimization of enzyme cocktails in the context of biorefineries.

## Methods

### Preparation of bioinspired assemblies

**Fig. 8:**
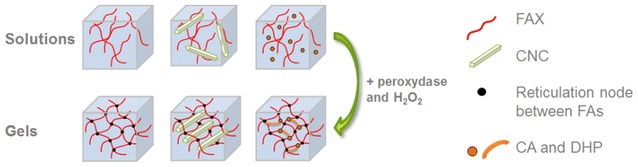
Schematic illustration of the bioinspired assemblies

Three types of polymers were used to prepare the bioinspired assemblies: hemicellulose as the central scaffold, crystalline cellulose, and DHP as model lignin (Fig. 8). The hemicellulose is FAX extracted from maize (Cambridge Biopolymers Ltd, Cambridge, UK) and contains 0.4 % FA [27]. CNC is prepared by sulfuric hydrolysis from ramie fibers [27]. Both polymers are solubilized in 50 mM phosphate buffer pH 6.5. Addition of an oxidative system consisting of peroxidase (Sigma, reference P8375) and hydrogen peroxide (Sigma, reference 216763) makes the FAX cross-link through inter- and intra-covalent interactions to turn the FAX solution into a hydrogel [19, 27]. CNCs can be added before triggering gelation, and if coniferyl alcohol (CA) is also mixed, the oxidative system can polymerize CA into dehydrogenation polymers (DHP). This is analogous to the Zulaufverfahren (ZL) procedure where all reactants are added simultaneously. Preparation of CA was done as previously described [33] and CA was mixed in dimethyl sulfoxide (DMSO). CA was chosen because guaiacyl units are the most abundant monomers of lignin in soft wood and are as abundant as syringyl units in hard wood [47]. Assemblies are prepared at room temperature by adding the different solutions of FAX, CNC, and CA at a selected concentration using the phosphate buffer and maintaining a DMSO concentration of 10 % (v/v). Oxidative system is added so that final concentrations of peroxidase and hydrogen peroxide are 17 µg/mL and 0.8 mg/mL, respectively.

### Characterization of bioinspired assemblies

#### Fluorescence spectroscopy

Mix of polymers was directly poured in a quartz cuvette to measure fluorescence before, during, and after gelation, in a spectrofluorimeter equipped with a Peltier temperature-control system (Jasco FP-8300, Tokyo, Japan). Fluorescence excitation range was set to 240–500 nm in order to follow fluorescence emission in the range 250–600 nm, using the same following parameters for all the samples: excitation and emission bandwidth of 2.5 nm and scan speed of 1000 nm/min. As a result, a 3D fluorescence spectrum of intensity Vs excitation and emission was obtained.

#### Infrared spectroscopy

Samples of bioinspired assemblies were frozen then freeze-dried. 2 mg of the resulting powder was mixed with 300 mg of KBr and a pellet of 1.5–2.0 mm thickness was pressed. Each pellet was analyzed in a Fourier transform infrared (FT-IR) spectrometer (Thermo FTIR Nicolet 6700). Acquisitions were averaged from 16 scans in transmission from 4000 to 400 cm^−1^ with noise correction. Spectra were analyzed in Omnic 8.1; baseline was corrected and normalization was performed using the area under the spectrum analyzed.

### Preparation of fluorescent probes

**Fig. 9:**
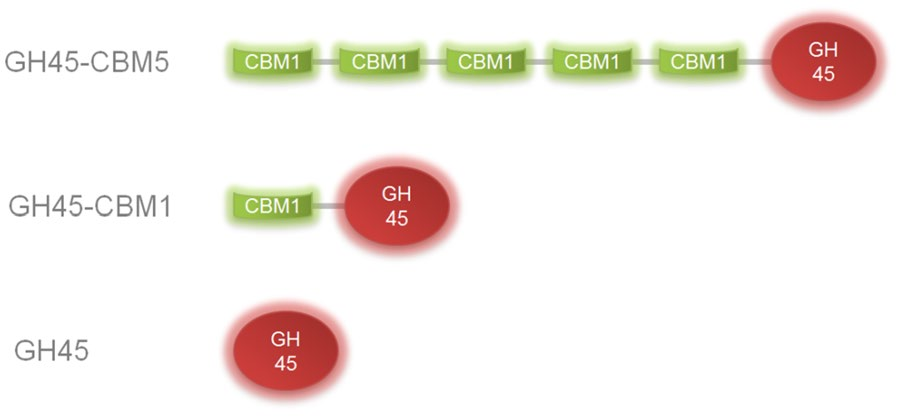
Schematic representation of the modularity of the three GH45-derived fluorescent probes

Three different enzymes were prepared as described in [29] based on the parental GH45-CBM5, which is composed of a catalytic domain and 5 CBMs in tandem at the *N*-terminal position (Fig. 9 and Additional file 4: Fig S4). The different probes were produced in *P. pastoris* and purified to homogeneity as described in [29].

#### Fluorescent GH45 probes: conjugation of proteins with FITC

Covalent grafting of fluorescein isothiocyanate (FITC) onto the different proteins can only occur with amine functions of the *N*-terminal residue and/or Lys residues. Analysis of the amino acid sequence of each protein shows that all they have several Lys residues: four for GH45 and GH45-CBM1, five for GH-45-CBM5 (Additional file 4: Fig S4). Structural analysis of the positions of the *N*-terminal residues and Lys residues based on homologous structure (data not shown) demonstrates that they are on the side opposite to the ligand binding site, suggesting that labeling would minimally or not at all affect the binding properties of the proteins.

The grafting experimental conditions are those previously used [28]. In order to get homogeneous fluorescent probes and to limit the impact of the presence of the fluorophore on enzymatic activity, conjugation molar ratio between proteins and FITC was adapted so that it was a constant value close to 1.0.

### Characterization of fluorescent probes

#### Enzymatic activity with/without FITC

Endoglucanase activity assayed using carboxy-methyl cellulose as substrate was in agreement with the previous study [29]. Comparative analysis of the different fluorescent probes with and without FITC revealed no significant differences in terms of endoglucanase activity (not shown).

#### Molecular weight and hydrodynamic radius

Absolute MW and hydrodynamic radius (*R*_H_) of the probes were determined by SEC-MALS-QELS. To summarize, 150 µL of each probes in 50 mM sodium nitrate buffer were injected at 0.6 mL/min on a Shodex KW 802.5 column equilibrated at 30 °C connected to the HPLC system (Waters 717), equipped as follows: degas, UV-visible detector (Waters 2996), multi-angle static light scattering (MALS) detector DAWN HELEOS II (Wyatt, Santa-Barbara, USA), dynamic light scattering detector DynaPro NanoStar (Wyatt), and refraction index detector (Waters 2414). Analysis of the chromatogram was performed with the ASTRA 6.1 software (Wyatt). Properties of GH45 fluorescent probes are summarized in Table 1.

### Mobility and interaction of fluorescent probes by fluorescence recovery after photobleaching (FRAP) in bioinspired assemblies

The mobility of the fluorescent probes was carried out in a confocal laser scanning microscope using the FRAP protocol previously described at room temperature [27]. In these conditions, the enzymatic activity of the probes on CNC was negligible and did not impact the CNC surface. At least six measurements were performed for each probe in each bioinspired assembly. As a result, two types of data were obtained: the experimental diffusion coefficient *D*_exp_ and the mobile fraction (MF) defined as the probe fraction that contributes to the fluorescence recovery. In the case *D*_exp_ being smaller than expected on the basis of the diffusing probe size, *D*_exp_ is called an effective diffusion constant [30]. The binding parameters can be determined by calculating the pseudo-affinity (or apparent affinity) constant ${{k_{{_{\text{on}} }}^{ * } } \mathord{\left/ {\vphantom {{k_{{_{\text{on}} }}^{ * } } {k_{\text{off}} }}} \right. \kern-0pt} {k_{\text{off}} }}$ from

1 $$\frac{{k_{\text{on}}^{ * } }}{{k_{\text{off}} }} = \frac{{D_{\text{ref}} - D_{\exp } }}{{D_{\exp } }},$$

where *D*_ref_ is the diffusion coefficient of the probe measured in buffer and *D*_exp_ is the diffusion coefficient of the same probe measured in a given assembly.

### Modeling fluorescent probe interactions

For each fluorescent probe, the pseudo-affinity constants calculated are considered as experimental data responses (values + standard errors). They were computed in a full factorial experiment designed in Design Expert 8.0 (Stat-Ease, Minneapolis, USA) using three principal factors *X*_*n*_ (*n* = 1 − 3) which correspond to the varied parameters. All possible coefficients A, B, and C and interaction coefficients AB, AC, AB, and ABC were also calculated, using a first-degree model equation. The same statistical analysis as previously done [27, 28] allows a quantification of the F-values of each coefficient, which defines the impact of each coefficient on the response (the pseudo-affinity) so that they can be hierarchized.

## Additional files

10.1186/s13068-016-0428-y F-values of principal coefficients and their interactions for each GH45 probe in FAX/CNC assemblies. A: probe type, B: FAX concentration, C: CNC concentration and their interactions AB, AC, BC, ABC.
10.1186/s13068-016-0428-y F-values of principal coefficients and their interactions for each GH45 probe in FAX/DHP assemblies. A: probe type, B: FAX concentration, C: DHP concentration and their interactions AB, AC, BC, ABC.
10.1186/s13068-016-0428-y F-values of principal coefficients and their interactions for each GH45 probe in FAX/CNC/DHP assemblies. A: probe type, B: FAX concentration, C: DHP concentration and their interactions AB, AC, BC, ABC.
10.1186/s13068-016-0428-y Amino acid sequences of GH45, GH45-CBM1 and GH45-CBM5. Lys residues are highlighted in blue, Trp in the catalytic domain are in green and Trp in the CBMs are in red.

## Abbreviations

CA: coniferyl alcohol
CBM: carbohydrate-binding module
CNC: cellulose nanocrystal
DHP: dehydrogenation polymer
FA: ferulic acid
FAX: feruloylated arabinoxylan
FRAP: fluorescence recovery after bleaching
GH: glycoside hydrolase
LCC: lignin–carbohydrate complex
MW: molecular weight
