# Supplementary material for: Investigation of the binding properties of a multi-modular GH45 cellulase using bioinspired model assemblies
Source: Biotechnol Biofuels. 2016 Jan 19;9:12. doi: 10.1186/s13068-016-0428-y (PMC4717654; doi:10.1186/s13068-016-0428-y)
Supplement: Supplementary file 1 — 10.1186/s13068-016-0428-y F-values of principal coefficients and their interactions for each GH45 probe in FAX/CNC assemblies. A: probe type, B: FAX concentration, C: CNC concentration and their interactions AB, AC, BC, ABC. [file 13068_2016_428_MOESM1_ESM.docx]

Figure S1. F-values of principal coefficients and their interactions for each GH45 probe in FAX/CNC assemblies. A: probe type, B: FAX concentration, C: CNC concentration and their interactions AB, AC, BC, ABC.
