# Supplementary material for: Investigation of the binding properties of a multi-modular GH45 cellulase using bioinspired model assemblies
Source: Biotechnol Biofuels. 2016 Jan 19;9:12. doi: 10.1186/s13068-016-0428-y (PMC4717654; doi:10.1186/s13068-016-0428-y)
Supplement: Supplementary file 4 — 10.1186/s13068-016-0428-y Amino acid sequences of GH45, GH45-CBM1 and GH45-CBM5. Lys residues are highlighted in blue, Trp in the catalytic domain are in green and Trp in the CBMs are in red. [file 13068_2016_428_MOESM4_ESM.docx]

**GH45**

GDFETIPNGFSGTGRTTRY**W**DCC**K**PSCS**W**PG**K**SNSVTGPVRSCGVSGNVLDANAQSGCIGGEAFTCDEQQP**W**SINDDLAYGFAAASLAGGSEDSSCCTCM**K**LTFTSSSIAG**K**TMIVQLTNTGADLGSNHFDIALPGGGLGIFTEGCSSQFGSGYQ**W**GNQYGGISSLAECDGLPSELQPGCQFRFG**W**FENADNPSVEFEQVSCPPEITSITGCARTDE

**GH45-CBM1**

DVPL**W**GQCGGIGYTGSTTCSQGSCVYLND**W**YFQCLPEEETTSSTSSSSSSSSSSTSSASSTSSTSSTSSTSSTSSSTSSSSIPTSTSSSGDFETIPNGFSGTGRTTRY**W**DCC**K**PSCS**W**PG**K**SNSVTGPVRSCGVSGNVLDANAQSGCIGGEAFTCDEQQP**W**SINDDLAYGFAAASLAGGSEDSSCCTCM**K**LTFTSSSIAG**K**TMIVQLTNTGADLGSNHFDIALPGGGLGIFTEGCSSQFGSGYQ**W**GNQYGGISSLAECDGLPSELQPGCQFRFG**W**FENADNPSVEFEQVSCPPEITSITGCARTDE

**GH45-CBM5**

AQAETASLYHQCGGAN**W**EGATQCISGAYCQSQNPYYYQCVATS**W**GYYTNTSISSTATLPSSSTTVSPTSSVVPTGLVSPLYGQCGGQN**W**NGATSCAQGSYC**K**YMNNYYFQCVPEADGNPAEISTFSENGEIIVTAIEAPT**W**AQCGGHGYYGPTKCQVGTSCRELNA**W**YYQCIPDDHTDASTTTLDPTSSFVSTTSLSTLPASSETTIVTPTSIAAEQVPL**W**GQCGGIGYTGSTICEQGSCVYLND**W**YYQCLISDQGTASTTSATTSITSFNVSSSSETTVIAPTSISTEDVPL**W**GQCGGIGYTGSTTCSQGSCVYLND**W**YFQCLPEEETTSSTSSSSSSSSSSTSSASSTSSTSSTSSTSSTSSSTSSSSIPTSTSSSGDFETIPNGFSGTGRTTRY**W**DCC**K**PSCS**W**PG**K**SNSVTGPVRSCGVSGNVLDANAQSGCIGGEAFTCDEQQP**W**SINDDLAYGFAAASLAGGSEDSSCCTCM**K**LTFTSSSIAG**K**TMIVQLTNTGADLGSNHFDIALPGGGLGIFTEGCSSQFGSGYQ**W**GNQYGGISSLAECDGLPSELQPGCQFRFG**W**FENADNPSVEFEQVSCPPEITSITGCARTDE

Figure S4. Amino acid sequences of GH45, GH45-CBM1 and GH45-CBM5. Lys residues are highlighted in blue, Trp in the catalytic domain in green and Trp in the CBMs in red.
